# A First Comprehensive Baseline of Hydrocarbon Pollution in Gulf of Mexico Fishes

**DOI:** 10.1038/s41598-020-62944-6

**Published:** 2020-04-15

**Authors:** Erin L. Pulster, Adolfo Gracia, Maickel Armenteros, Gerardo Toro-Farmer, Susan M. Snyder, Brigid E. Carr, Madison R. Schwaab, Tiffany J. Nicholson, Justin Mrowicki, Steven A. Murawski

**Affiliations:** 1 University of South Florida, 140 7th Avenue South, St. Petersburg, FL 33701 USA; 2 Universidad Nacional Autónoma de México, Instituto de Ciencias del Mar y Limnología, Ciudad de México, CDMX México; 3 Universidad de La Habana, Centro de Investigaciones Marinas, 16 # 114, Playa, Habana, 11300 Cuba; 4 New College of Florida, 5800 Bay Shore Road, Sarasota, FL 34243 USA

**Keywords:** Environmental chemistry, Environmental monitoring, Marine chemistry

## Abstract

Despite over seven decades of production and hundreds of oil spills per year, there were no comprehensive baselines for petroleum contamination in the Gulf of Mexico (GoM) prior to this study. Subsequent to the 2010 *Deepwater Horizon* (DWH) spill, we implemented Gulf-wide fish surveys extending over seven years (2011–2018). A total of 2,503 fishes, comprised of 91 species, were sampled from 359 locations and evaluated for biliary polycyclic aromatic hydrocarbon (PAH) concentrations. The northern GoM had significantly higher total biliary PAH concentrations than the West Florida Shelf, and coastal regions off Mexico and Cuba. The highest concentrations of biliary PAH metabolites occurred in Yellowfin Tuna (*Thunnus albacares*), Golden Tilefish (*Lopholatilus chamaeleonticeps*), and Red Drum (*Sciaenops ocellatus*). Conversely, biliary PAH concentrations were relatively low for most other species including economically important snappers and groupers. While oil contamination in most demersal species in the north central GoM declined in the first few years following DWH, more recent increases in exposure to PAHs in some species suggest a complex interaction between multiple input sources and possible re-suspension or bioturbation of oil-contaminated sediments. This study provides the most comprehensive baselines of PAH exposure in fishes ever conducted for a large marine ecosystem.

## Introduction

**Figure 1:**
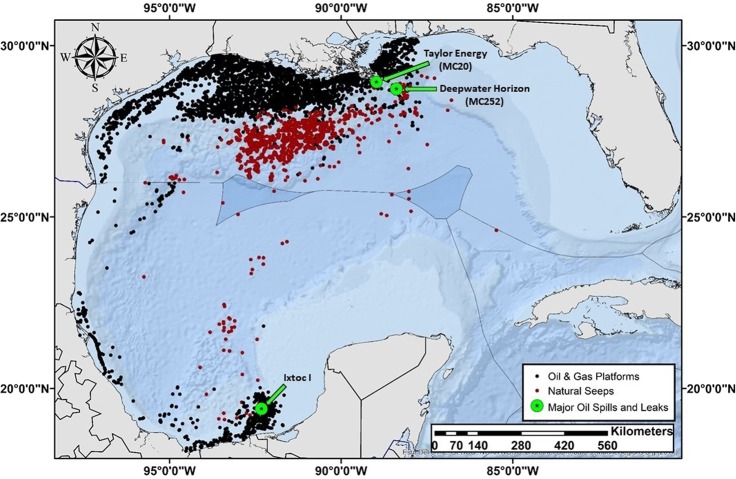
Oil and gas infrastructure and natural oil seeps in the GoM. The 1979 Ixtoc 1, 2004 Taylor Energy’s MC20 leak and the 2010 Deepwater Horizon oil blowouts are indicated. Seep shapefiles were provided by MacDonald *et al*.^44^. Shapefiles for the USA and Mexican oil platforms were downloaded from the Bureau of Ocean Energy Management (www.boem.gov) and the Centro Nacional de Información de Hidrocarburos (CNIH; mapa.hidrocarburos.gob.mx), respectively. This figure was generated in ArcGIS Desktop Version 10.5.1.

The Gulf of Mexico (GoM) is a large semi-enclosed basin connected to the Atlantic Ocean and Caribbean Sea through the Florida Straits and Yucatán Channel^1^. The marine oil and gas industry in the GoM was initiated in northern and southern Gulf during 1938 and 1950, respectively. By 2018, the oil and gas industry rapidly expanded to encompass over 5.3 million hectares in U.S. waters leaving the northern GoM littered with infrastructure consisting of more than 10,000 platforms and active rigs, 68,000 kilometers of active and inactive pipelines, and 27,000 abandoned wells. In the southern GoM there are more than 2,000 offshore wells and thousands of miles of pipelines extending south along the eastern state of Veracruz to the Bay of Campeche (Fig. 1).

Although there are thousands of oil and gas infrastructures in the GoM, historical reports suggest that approximately 5 percent of the oil discharged in North American waters is related to the extraction and transportation of petroleum compared to natural seeps (32%) and petroleum consumption (60%; land-based run-off, marine vessels, atmospheric deposition, aircraft fuel, non-tank spills and operational discharge)^2^. Nonetheless, the oil extraction and transportation processes often result in spills of varying magnitudes. For instance, between 1964 and 2012 there were 343 oil spills (>50 barrels) along the Outer Continental Shelf (OCS) of the GoM compared to the seven OCS oil spills recorded in the Pacific region during the same period (www.bsee.gov). In addition, more than 44 major oil spills (>200 barrels) and thousands of minor oil spills (<50 barrels) occur annually in U.S. waters^3–8^. The two largest oils spills in the GoM’s history were the 1979 *Ixtoc 1* spilling approximately 3 million barrels of oil and the 2010 *Deepwater Horizon* (DWH), spilling 4.9 million barrels of oil^9,10^.

The imminent questions following any major accident or oil spill often pertain to the associated ecosystem impacts and length of the recovery period. Historical measurements or baseline data collected in the months or years prior to the spill are critical in answering such questions. Characterizing ecosystem recovery is a multifaceted task involving both complex temporal and spatial processes, which can drastically differ depending on the scale, variable, and species being monitored^11–13^. A number of challenges and ecological assumptions are incorporated in the recovery assessment process, including the classification of the preexisting ecosystem state, selecting reference locations that are similar both oceanographically and environmentally to impacted site(s), and the availability of valid historical baseline data.

Recovery assessments following oil spills, such as the *Exxon Valdez, Ixtoc 1* or DWH, can be relatively straight-forward where recovery is defined as occurring when the conditions being measured return to similar conditions of those prior to the incident (e.g., return to baseline)^13^. Yet, subsequent to all three of these spills, the dearth of baseline data and identifying unimpacted reference sites, has hindered impact assessments for decades^1,4,5,7,14^. Assessing recovery, the natural variability, and the overall environmental health of the GoM is especially complicated by the extensive expansion of the offshore oil and gas industry combined with the multiplicity of routine inputs of an admixture of polycyclic aromatic hydrocarbons (PAHs) and multiple simultaneous stressors from natural (e.g., seeps) and anthropogenic contaminant sources^15–17^. It is still somewhat perplexing that the lack of baseline data remains a recurrent problem in the GoM after seven decades of oil and gas exploration and expansion.

There was a massive push by the scientific community to generate critical data needed to fill knowledge gaps and understand the consequential impacts from the DWH spill. Subsequent studies have documented multiple impacts on the ecosystem extending from coastal marshes to the deep sea^18–20^. Impacts on fisheries included changes in population dynamics, diet, trophic level, disease frequency and physiological output, but it could take decades for the impacts to be fully expressed^21^.

Polycyclic aromatic hydrocarbons (PAHs) are considered the most toxic component of oil yet baseline data measuring oil exposures and tissue levels of PAHs in GoM fish prior to DWH are scarce. Measuring PAHs in fish has been largely ignored due to the underlying assumption that PAHs generally do not bioaccumulate in fish. This assumption disregards the impacts of chronic, long-term exposures that lead to increased body burdens and related toxicities. Fish have a well-developed metabolic system capable of efficiently converting PAHs into water soluble compounds that are stored in the bile for excretion^22^. The bile is emptied into the gastrointestinal tract where PAHs and associated metabolites can become reabsorbed, recirculated, and recycled between the liver, bile, and gastrointestinal tract^23^. This recirculating reservoir of PAHs amplifies the potential for binding to proteins and genetic material (e.g., DNA and RNA). Although fish have efficient biotransformation capabilities, they do not have a highly developed DNA repair system, thus promoting the susceptibility to adverse toxic reactions including lesions, mutagenesis, teratogenesis, and carcinogenesis^24^. These processes emphasize the use of biliary PAH metabolites as sensitive indicators and key biomarkers for assessing ongoing and recent (days to weeks) exposures to oil and PAHs in fish^25–28^.

We conducted comprehensive, systematic, Gulf-wide fish surveys using commercial fishing vessels and the *R/V Weatherbird II* over seven years, 2011–2018. Data were used to evaluate spatial differences in fish composition and diversity^29^, changes in populations and growth rates^30^, disease frequency and contaminant levels^28,31,32^, and health and immune responses in fishes^33,34^. In this study, we quantified biliary PAH metabolite concentrations in fishes collected Gulf-wide in order to (1) assess post-DWH PAH concentrations; (2) understand temporal and spatial exposure differences among species and regions; and (3) monitor long-term trends (2011–2017) and environmental changes in the north central GoM, with an overarching goal of producing ecological baselines for environmental preparedness. While it is beyond the scope of this article to discuss all 91 species collected individually and in depth, select species (e.g., Tilefish, Hake, Grouper) have been further discussed in detail in previous manuscripts^3,35,36^. Instead, the main focus of this research is to provide baseline information and characterize the relative regional concentrations of PAHs in fish collected in the Gulf of Mexico.

## Results and Discussion

### Regional spatial patterns

**Figure 2:**
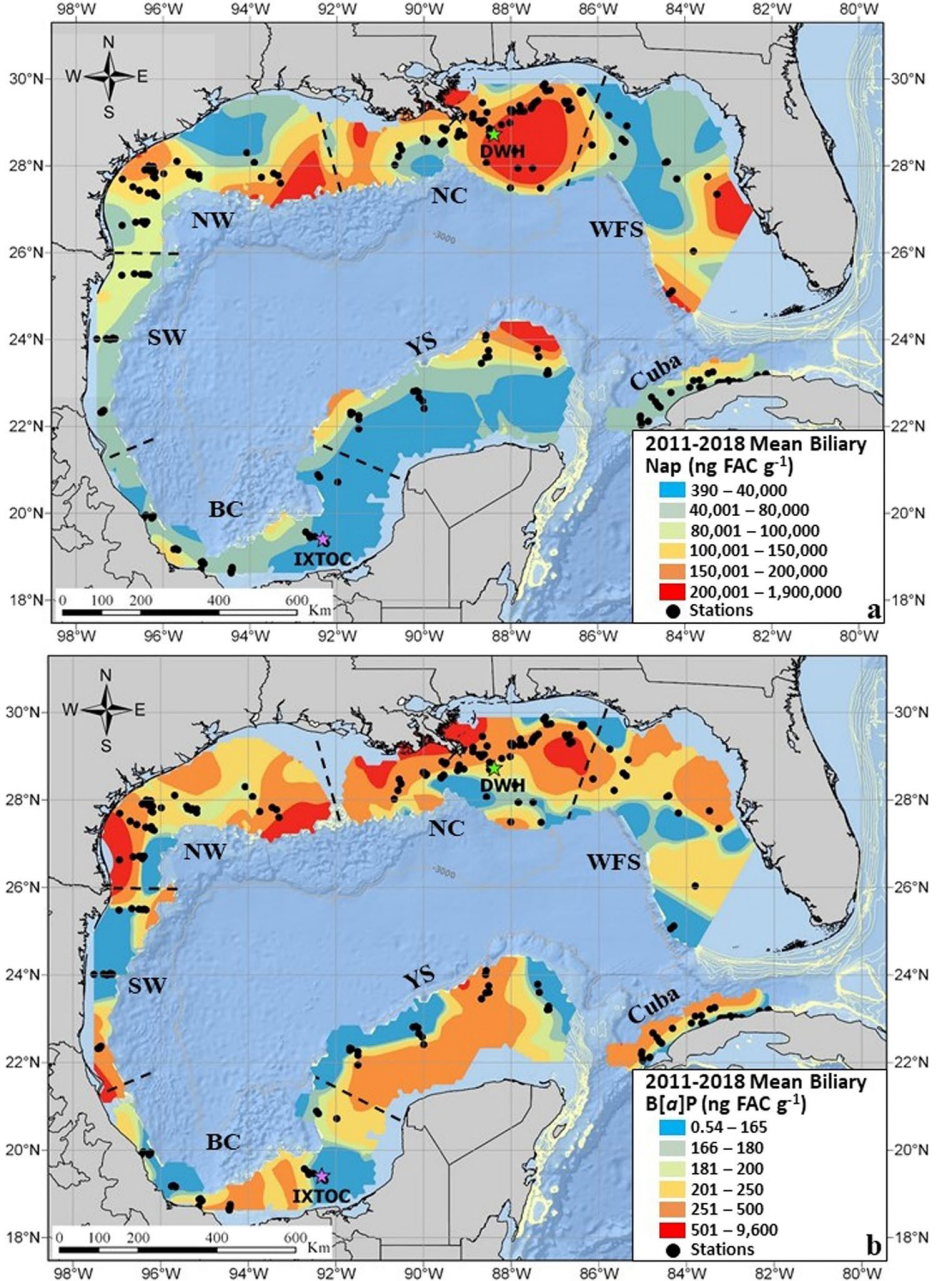
Spatial concentration maps for mean (**a**) biliary naphthalene (Nap, ME = 0.012, RMSE = 0.95) and (**b**) benzo[*a*]pyrene (B[*a*]P, ME = 0.004, RMSE = 1.02) equivalents (ng FAC g^−1^ bile) in fish (n = 2,503) collected from coastal, continental shelf and deep-sea areas in the GoM, 2011–2018. Region designations are the Bay of Campeche (BC), Cuba, north central (NC), northwest (NW), southwest (SW), West Florida Shelf (WFS) and the Yucatán Shelf (YS). Spatial concentration maps were generated using the Kernel Interpolation with Barriers method in ArcGIS Pro Version 2.3.

Biliary PAH metabolites were measured in 2,503 fish (91 species) occupying pelagic, demersal (benthic), reef and inshore habitats. Significant regional differences in biliary total PAH equivalents (TPAHeqs) were identified in the GoM for all years and species combined (2011–2018, *F* = 45.7, *p* = 0.001). Combining all species together within the same contour map are primarily meant to convey the relative concentrations PAH exposures in GoM fish. Mean biliary TPAHeqs measured in fish collected from the north central (NC) region of the GoM were significantly higher (*p* < 0.001) than all other regions in the Gulf (Table 1, Fig. 2). The next highest concentrations were measured in the northwest, followed by the Yucatán Shelf, southwest region, West Florida Shelf, and Cuba. The lowest levels of mean TPAHeqs were measured in fish from the Bay of Campeche, an area known for persistent and extensive oil slicks resulting from oil spills (56%) and natural seeps (44%)^37^. The highly productive Cantarell Oil Field Complex and active natural oil seeps (e.g, Cantarell Oil Seep cluster) are located in this region. In 1979, the *Ixtoc 1* platform within the Cantarell Oil Complex experienced a subsurface blowout that leaked for more than 9 months; ultimately releasing ~3 million barrels of oil into the Bay of Campeche and southern GoM^10^. The narrow shelf and large wave action in the southwestern GoM may result in the continuous flushing and removal of oil contamination and thus the reduction of chronic exposures in this region^38,39^.

**Table 1:** Mean, median and range of biliary naphthalene, benzo[a]pyrene and total PAH equivalent (TPAHeq) concentrations (ng FAC g^−1^ bile) in fish by region and year in the Gulf of Mexico, 2011–2018.

Regions	Collection Year(s)	*n*	Naphthalene			Benzo[*a*]pyrene			TPAHeq		
			Mean	Median	Range	Mean	Median	Range	Mean	Median	Range
Bay of Campeche (BC)	2015	125	50,000	20,000	410–550,000	190	130	0.54–1,400	50,000	20,000	430–550,000
	2016	120	60,000	33,000	880–380,000	160	120	19–730	60,000	33,000	1,000–380,000
	2015–2016	245	55,000	26,000	410–550,000	180	120	0.54–1,400	55,000	26,000	430–550,000
Cuba (CUB)	2017	225	70,000	48,000	390–350,000	200	160	0.92–3,200	70,000	48,000	450–350,000
North central (NC)	2011	34	130,000	110,000	41,000–470,000	280	280	94–590	130,000	110,000	41,000–110,000
	2012	135	140,000	79,000	11,000–680,000	460	260	46–2,000	140,000	80,000	11,000–680,000
	2013	199	98,000	51,000	2,800–480,000	310	200	10–1,900	98,000	51,000	2,800–480,000
	2014	179	140,000	42,000	600–1,100,000	400	180	8.1–4,600	140,000	42,000	630–1,100,000
	2015	260	130,000	33,000	480–1,900,000	300	120	0.74–3,900	130,000	34,000	480–1,900,000
	2017	194	270,000	120,000	6,800–1,800,000	300	120	0.99–8,600	270,000	120,000	7,000–1,800,000
	2018	37	250,000	110,000	32,000–1,300,000	220	110	7.2–1,400	250,000	110,000	32,000–1,300,000
	2011–2018	1,038	160,000	63,000	480–1,900,000	340	170	0.74–8,600	160,000	64,000	480–1,900,000
Northwest (NW)	2016	349	110,000	73,000	2,800–830,000	280	160	8.9–4,600	110,000	73,000	3,000–830,000
	2017	42	220,000	190,000	10,000–1,000,000	240	180	30–770	220,000	190,000	11,000–1,000,000
	2016–2017	391	120,000	79,000	2,800–1,000,000	270	160	8.9–4,600	120,000	79,000	3,000–1,000,000
Southwest (SW)	2016	129	82,000	55,000	2,000–710,000	290	110	4.4–9,600	82,000	55,000	2,000–710,000
West Florida Shelf (WFS)	2013	38	54,000	21,000	1,400–360,000	280	110	18–3,000	54,000	21,000	1,500–360,000
	2014	33	33,000	21,000	4,400–160,000	190	160	33–470	33,000	21,000	4,500–160,000
	2015	21	13,000	8,200	3,600–45,000	130	100	25–360	13,000	8,400	3,600–45,000
	2017	57	150,000	120,000	8,600–580,000	220	140	22–2,200	150,000	120,000	10,000–580,000
	2013–2017	278	80,000	30,000	1,400–580,000	220	140	18–3,000	80,000	30,000	1,500–580,000
Yucatán Shelf (YS)	2015	92	48,000	15,000	470–350,000	170	99	4.3–950	48,000	15,000	470–350,000
	2016	234	100,000	62,000	4,000–960,000	230	140	9.2–3,900	100,000	62,000	4,100–960,000
	2015–2016	326	85,000	50,000	470–960,000	210	130	4.3–3,900	85,000	50,000	470–960,000
Gulf of Mexico Mean	2011–2018	2,503	120,000	54,000	390–1,900,000	270	150	0.54–9,600	120,000	54,000	430–1,900,000

*n* = sample size.

TPAHeq = sum of naphthalene and benzo[*a*]pyrene equivalent concentrations.

This regional trend was also observed within a particular year where multiple regions were sampled. In 2013 (*F* = 8.13, *p* = 0.001), 2015 (*F* = 22.5, *p* = 0.001) and 2017 (*F* = 34.7, *p* = 0.001) biliary TPAHeqs collected from fish in the north central region were significantly higher than other regions sampled in the same year. In 2013, the mean biliary TPAHeqs in fish from the north central region were nearly double those in the West Florida Shelf (*p* = 0.014). Mean biliary TPAHeqs in the north central region in 2015 were an order of magnitude higher than the West Florida Shelf (*p* = 0.001), 1.5x higher than the Yucatán Shelf (*p* = 0.001) and almost three times higher than the Bay of Campeche (*p* = 0.001). The 2017 biliary TPAHeqs in fish from the north central region were two- and four-times higher than fish collected along the West Florida Shelf (p = 0.002) and Cuba (*p* = 0.001), respectively, yet similar to those collected in the northwest region (*p* = 0.19).

The contour maps of mean biliary naphthalene (Fig. 2a) and benzo[a]pyrene (Fig. 2b) metabolite concentrations provide a broad-scale baseline for PAH concentrations across the GoM continental shelves and in some deep-sea areas off the north central region. The “hot spots” of PAH exposure provide important insights to possible sources of these PAHs in the GoM. In aquatic environments, PAHs primarily originate from petrogenic and pyrogenic sources and to a lesser degree, diagenetic and biogenic sources^40^. Petrogenic sources are the low molecular weight (2–3 aromatic rings, LMW) PAHs created by diagenetic processes at relatively low temperatures and are typically associated with local or point sources, such as oil platforms, refineries, other petroleum industries, vehicle exhaust, evaporated gasoline, diesel fuel, and boat discharge. For example, there are relatively high concentrations of the LMW PAH metabolite, naphthalene, in the northwestern region, the north central region, off the central West Florida Shelf, and the Yucatán Shelf (Fig. 2a). These locations are either near major population centers (e.g., Tampa Bay, Florida), along marine traffic thoroughfares (e.g Yucatán Shelf), or within an area of high density oil and gas infrastructure (e.g., northwest and north central region). The large hotspot of mean naphthalene equivalent concentrations in the north central region is likely a combination of the resuspension of DWH contaminated sediments, additional oil spills, leaking wells (e.g., Taylor Energy’s Saratoga platform MC20), and riverine inputs from the Mississippi River Delta^41,42^. The naphthalene hotspot off the northern tip of the Yucatán Shelf is somewhat enigmatic as it is located far from both coastal populations and the extant Mexican oil infrastructure. There are however a number of natural seeps offshore (>3,500 m) of the northern Yucatán Shelf, as well as high rates of vessel traffic, which could potentially be contributing to the elevated PAH contamination in this region^43–46^. Another contributing factor to the hotspots off the Yucatán Shelf and Tampa Bay could also be the presence of submarine groundwater discharge zones which can have a significant influence on environmental conditions in coastal marine environments^47^.

Pyrogenic sources are made up of the high molecular weight (4–6 aromatic rings, HMW) PAHs generally associated anthropogenic pollution and are formed by the incomplete short duration combustion of various fossil fuels (such as oil, gas, coal, and vehicle emissions) and organic matter (such as forest fires and volcanoes) at high temperatures. Relatively high concentrations of the HMW PAH, benzo[*a*]pyrene, were observed adjacent to the Rio Grande and the Mississippi River Delta, as well as relatively elevated concentrations along the northern coast of Cuba (Fig. 2b). Contributing factors in these areas may be their proximity to anthropogenic sources associated with the petroleum industry, various coastal inputs (e.g., terrestrial run-off, riverine discharge) and marine navigational routes.

### Temporal patterns

The longest time series with repeat fish surveys was conducted in the north central region (2011–2015, 2017–2018) of the GoM post-DWH, therefore, temporal trends were only evaluated in this region (Table 1). Fish surveys in the NC consisted of both demersal (2011–2015, 2017) and pelagic longlines (2018). To avoid collection method as a confounding factor the 2018 pelagic survey was excluded from the temporal trend analysis.

Significant temporal trends (*F* = 12.3, *p* = 0.001) in mean biliary TPAHeq concentrations for all fishes combined were identified in the north central (NC) region of the GoM over a seven-year period (2011–2015, 2017, Fig. 3). Mean concentrations of biliary TPAHeqs in 2017 were significantly higher than levels in 2011 (*p* = 0.002), 2012 (*p* = 0.001), 2013 (*p* = 0.001), 2014 (*p* = 0.001) and 2015 (*p* = 0.001). In general, biliary TPAH metabolites in the north central region varied over time with an initial 24% decrease during the first three years post-DWH, followed by a 173% increase by 2017 (*p* = 0.001).

**Figure 3:**
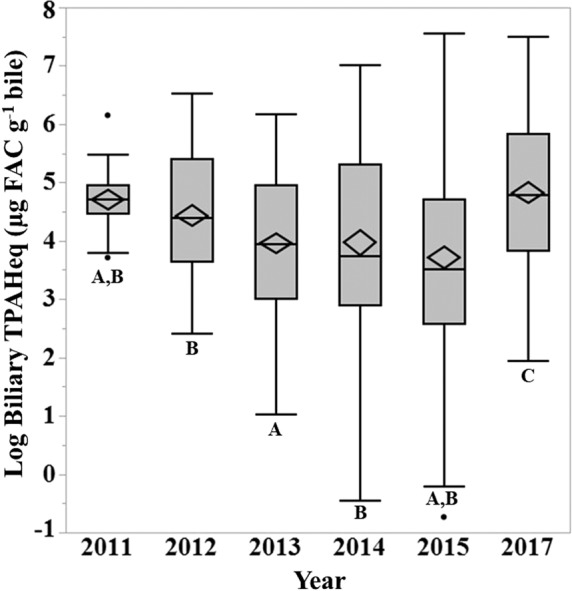
Box plots illustrating temporal trends observed in biliary PAH concentrations (µg FAC g^−1^ bile) in fish (all species combined) collected in the north central region of the GoM, 2011–2017. The line represents the median and the confidence diamond contains the mean and the upper and lower 95% confidence intervals of the mean. Years not connected by the same letter are statistically different.

A recent investigation reported significant declines in biliary PAHs within the first few years following DWH (2011–2015) for several species in the north central region^3,28^. The current study supplements previous reports by Pulster *et al*.^3^ and Snyder *et al*.^28^ by extending the time series through 2017. For example, King Snake Eel in the north central region demonstrated a 41% decrease in biliary PAHs between 2012 and 2015 (*p* = 0.04) and then experienced a 126% increase between 2015 and 2017 (*p* = 0.002). Biliary PAHs in Red Snapper declined 52% between 2011 and 2013 (*p* = 0.005) followed by a 141% increase between 2013 and 2017 (*p* = 0.027). Similarly, a recent study reported a 178% increase in biliary PAH concentrations for Golden Tilefish collected in the north central region between 2012 and 2017^35^. Between 2012 and 2014, the pattern of increasing PAHs has been observed in sediments, fishes and birds^3,48–53^. These increases have been attributed to the resuspension of contaminated sediments that often occur following natural disturbances (e.g., bioturbation, tropical storms and hurricanes, currents and waves)^41,52,53^. However, the ongoing release of oil and gas from the 2004 toppled Taylor Energy Company’s Saratoga oil platform (MC20)^42,54^ and the lingering leak of DWH until at least 2012^55^ cannot be dismissed as possible sources. Other contributing inputs could be from various sources including riverine input and the documented nine spills in this region between 2012 and 2013 followed by three additional spills between 2014 and 2017 (www.bsee.gov).

The only species with notable decreases in biliary PAHs over the entire time series were sharks. Biliary PAH concentrations have shown a continuous decline in Little Gulper Shark (*Centrophorus uyato*) over time, declining 83% between 2014 and 2017 (*p* = 0.49). A 67% decrease in biliary PAHs was also observed for Shortfin Mako (*Isurus oxyrinchus*) between 2012 and 2017 (*p* = 0.08). These species were collected at the same sites and depths as other species (e.g., Golden Tilefish, King Snake Eels, Hakes) that are demonstrating increasing levels of biliary PAHs over time. Research has suggested that migration and movement patterns may have influenced the extent and timing of exposures to DWH oil in some shark species^56^. In this study, the sample sizes for these species are too small to make any inferences but does warrant further investigation.

### Species comparisons

Games-Howell HSD post-hoc analyses identified a number of species-specific differences in the mean biliary TPAHeq dataset for all years and regions combined (2011–2018, Fig. 4, Table S2). Interpretation is complex as hydrocarbon uptake, metabolism, and excretion may be influenced by regional and species-specific elements such as diet, habitat, and physiology. The focus of this analysis is to provide broad-scale baseline data on exposure in the species present in a given environment, therefore, interpreting species-specific difference is not the main focus of this discussion.

**Figure 4:**
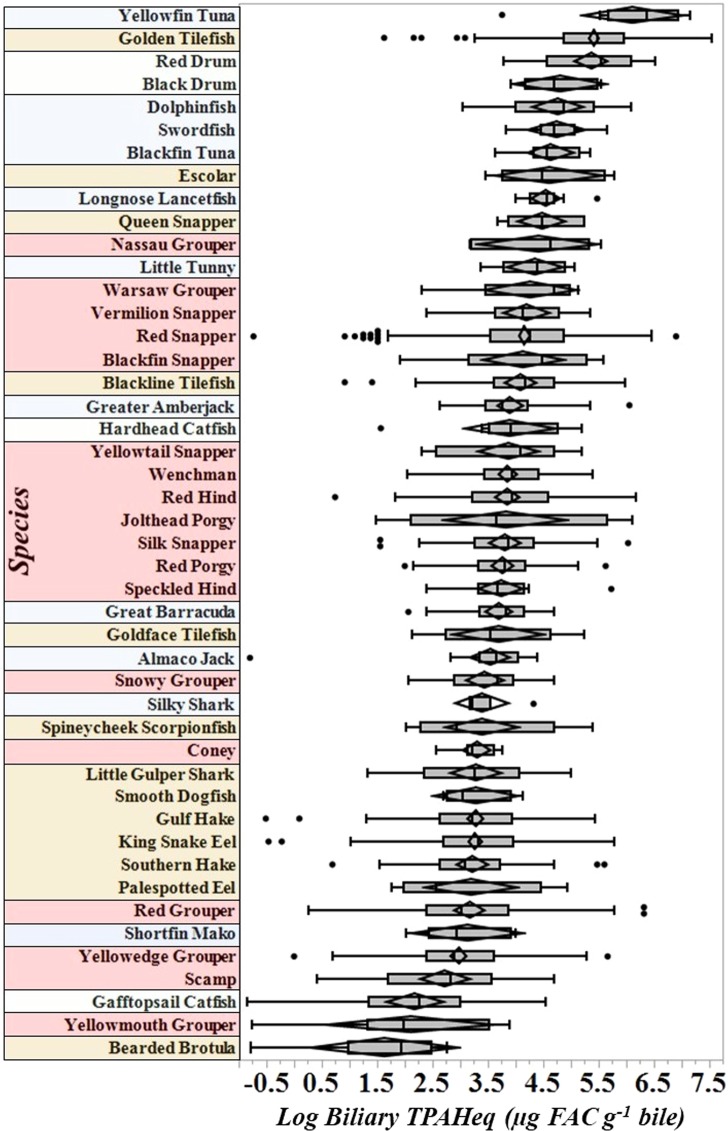
Biliary PAHs (µg FAC g^−1^ bile) measured in 46 of the 91 species of fish collected in the GoM (2011–2018). This figure is a representation of the range of biliary PAHs observed across pelagic, demersal, coastal inshore and benthic-reef associated species. Pelagic, demersal, coastal inshore, and benthic-reef associated species are highlighted in blue, brown, green and red, respectively. Concentrations are sorted in descending order of biliary PAH concentrations. Statistical differences between species can be found in Table S2.

Biliary PAHs metabolite equivalent concentrations for fish collected in the GoM ranged from 0.430 to 1,900 µg FAC g^−1^ bile (Table 1). The wide range of biliary PAH metabolites in fish collected in the GoM are within the same range as fish collected from polluted harbors and estuaries in Brazil (65.5–589 µg g^−1^ bile)^57^ and Chesapeake Bay (365 ± 203 µg NAP FAC g^−1^ bile)^58^ as well as those collected following the 1994 Columbia River (200 ± 52 µg NAP FAC g^−1^ bile)^59^ and 1989 *Exxon Valdez* (270–2,600 µg NAP FAC g^−1^ bile) oil spills^60^.

The highest mean biliary PAH levels were found in a diverse assortment of species, including the pelagic Yellowfin Tuna (*Thunnus albacore*, 640 ± 430 µg FAC g^−1^), Skipjack Tuna (*Katsuwonus pelamis*, 340 ± 92 µg FAC g^−1^), the demersal Golden Tilefish (320 ± 290 µg FAC g^−1^) and the inshore Red Drum (*Sciaenops ocellatus*, 280 ± 180 µg FAC g^−1^) (Fig. 4). The lowest concentrations were found in Bearded Brotula (*Brotula barbata;* 7.7 ± 5.4 µg FAC g^−1^), Remora (*Echeneidae sp*.) and Leopard Toadfish (*Opsanus pardus*). The Golden Tilefish, a demersal species sampled Gulf-wide, has been previously identified as having some of the highest levels of biliary PAHs measured globally^28^.

Biliary concentrations were compared by habitat type (pelagic, demersal, reef and coastal inshore) to investigate the influence of lifestyle (*F* = 46.5, *p* = 0.001). The pelagic species consisted of 26 species such as jacks, tuna, barracuda, sharks, and dolphinfish. Reef species (42 sp.) consisted of snappers, groupers, porgy, lionfish etc. Coastal inshore species (4 sp.) were mainly alligator gar, catfish and drum. Tilefish, hakes, flounder, eels, etc. primarily made up the demersal group (19 sp.). The demersal group (160,000 ± 240,000 ng FAC/g bile) had significantly higher biliary PAHs than the pelagic (100,000 ± 160,000 ng FAC/g bile, *p* = 0.002) and reef (75,000 ± 86,000 ng FAC/g bile, *p* = 0.001) species. The biliary PAHs in reef species were also significantly lower than both the pelagic (*p* = 0.031) and inshore (160,000 ± 180,000 ng FAC/g bile, *p* = 0.007) species. The coastal inshore species had similar biliary PAH levels as the pelagic species and demersal species.

While the demersal group had significantly higher biliary PAHs than the pelagic group, this was not universal when comparing individual species across habitat types. For example, Yellowfin Tuna and the Golden Tilefish had significantly higher biliary PAH concentrations than 24 and 31 other species, respectively, consisting of both pelagic (e.g., Barracuda, Amberjack and Shortfin Mako) and demersal (e.g., tilefish, groupers, snappers) species (Fig. 4, Table S2). Additionally, biliary PAHs in the inshore coastal species, Red Drum, had significantly higher levels than 33 species, including both inshore (e.g., catfish) and offshore demersal, reef and pelagic species (e.g., groupers, snappers, hakes, and jacks). The majority of Yellowfin Tuna were collected in 2018 from the north central region of the GoM but had similar levels (*p* > 0.05) as the demersal Golden Tilefish, collected Gulf-wide and the inshore species, Red Drum collected in coastal Louisiana. Demersal and inshore species are expected to have relatively higher levels of exposure due to associations with sediments and anthropogenic sources, respectively. It was unexpected to find relatively high levels of biliary PAHs in Yellowfin Tuna.

The majority of PAHs rapidly bind to suspended or resuspended particles and settle to the benthos. High concentrations of biliary PAHs were therefore expected in the Golden Tilefish based on their demersal and burrowing lifestyle. Yellowfin Tuna however are pelagic swimmers remaining in the upper 200 m of the water column, spending the majority of their time (72%) in the upper 50 m^61^. Water concentrations of PAHs vary considerably but are typically detected at trace levels or below detection limits since they generally dissipate quickly due to weathering processes (e.g., evaporation, photooxidation, dissolution and biodegradation)^62,63^. Theoretical possibilities for the high levels of PAHs in tuna may be attributed to high surface level sources (e.g., produced waters^64^) combined with increased gill uptake^65^ as a result of high swim speeds^66^.

### Biliary PAH correlations with physical and biological variables

Physical parameters, biometrics and health proxies were evaluated for relationships with biliary TPAHeqs. Previous evidence demonstrates the increase in persistence and exposure of PAHs in fish with decreasing temperatures (resulting in elevated biliary PAHs)^67,68^. Deep, cold water can reduce both the volatilization and biodegradation rates of PAHs and consequently increase exposure and tissue concentrations in fish. This may be primarily due to the impacts of temperature on the metabolic processes in aquatic organisms. For all fish in this study, very weak relationships were detected between biliary PAHs and water temperature (r = −0.22, *p* = 0.001) or depth (r = 0.07, *p* = 0.002). Although sampling in this study occurred across a relatively wide temperature range (17 ± 5 °C), the very weak correlations observed suggest that the temperature and depth range may not be large enough to elicit changes in physiochemical parameters. Additionally, temperature and depth may have relatively little impact in this region when compared to other highly species-specific biological and physiochemical variables affecting the bioavailability, uptake, biotransformation and excretion rates of PAHs^22,28,68,69^.

For all 91 species combined, relationships were explored between mean biliary TPAHeq concentrations and standard length, total weight, liver weight, gastrointestinal weight, condition factor (K), and trophic level (r = 0.042, *p* = 0.040) (Table 2). Weak (r = 0.30 to 0.50) to very weak (r < 0.30) positive relationships were detected between biliary PAHs and standard length, total weight, trophic level and condition factors. Laboratory studies have previously demonstrated both PAH metabolite concentration and bile volume are strongly influenced by the feeding status of fish, with both being the greatest in fish with empty stomachs^70^. The gastrointestinal weights were used as a proxy for feeding status in this study, yet they did not appear to be correlated with biliary PAHs (Table 2).

**Table 2:** Permutation-based correlations (Pearson’s, r) between biliary PAH concentrations and biometrics for all 91 species combined and select species with samples sizes (*n*) greater than 100.

Species	Statistic	Standard Length (cm)	Total Weight (kg)	Liver Weight (kg)	GI^a^ Weight (kg)	Condition Factor (K)
91 species combined^b^	r	0.180	0.096	−0.011	−0.001	0.0210
	*p-*value	0.001	0.001	0.628	0.957	0.353
	*n*	2,106	2,502	2,093	2,093	2,133
Golden Tilefish	r	0.086	0.073	0.019	0.112	0.171
	*p-*value	0.063	0.178	0.717	0.036	0.001
	*n*	430	391	351	350	389
Gulf Hake	r	−0.222	−0.209	−0.219	−0.116	−0.081
	*p-*value	0.004	0.009	0.011	0.131	0.184
	*n*	163	149	142	142	149
King Snake Eel^c^	r	−0.212	−0.157	−0.051	−0.070	−0.008
	*p-*value	0.001	0.015	0.427	0.367	0.894
	*n*	262	251	204	182	250
Red Snapper	r	−0.320	−0.285	−0.229	−0.203	0.029
	*p-*value	0.001	0.001	0.001	0.001	0.552
	*n*	468	458	383	380	312
Yellowedge Grouper	r	0.055	0.099	0.110	0.100	0.038
	*p-*value	0.527	0.208	0.15	0.242	0.634
	*n*	145	140	137	137	139

^a^GI = gastrointestinal.

^b^Biometrics for combined species were standardized using z-scores.

^c^Total lengths were used for King Snake Eels.

Samples sizes were too small (<10) for several species included in this dataset to further evaluate relationships for each species. There were similar relationships detected between mean biliary TPAHeq concentrations and biometrics for species with large sample sizes (*n* > 100), such as Golden Tilefish (*Lopholatilus chamaeleonticeps*), Gulf Hake (*Urophycis cirrata*), King Snake Eels (*Ophichthus rex*), Red Snapper (*Lutjanus campechanus*), and Yellowedge Grouper (*Hyporthodus flavolimbatus*) (Table 2). The strength of the relationships between biliary PAHs and biometrics appears to be species-specific. All significant correlations were considered weak to very-weak explaining less than 30% of the data. This implies that biometrics are unlikely to have a substantial influence on biliary PAH levels.

Biliary PAHs in all fish combined were significantly different between sexes. Male (*p* < 0.0001) fish had significantly higher biliary PAH concentrations compared to females. The lower biliary PAHs in females may potentially indicate females have higher biotransformation rates than males. Sex differences are more than likely driven by higher energy expenditure rates and swimming activity in males compared to females^71^. Additional biomarkers (e.g., EROD) would be needed to fully investigate the metabolic activity and biotransformation rates between sexes of each species.

These data add strong supportive evidence that PAHs do not behave like other persistent organic pollutants with comparable octanol-water partitioning coefficients and molecular weights. This is primarily due to the ability of fish to effectively metabolize PAHs, which results in low assimilation efficiencies within individual fish, and minimal transfer to higher trophic levels^72^. Previous studies have demonstrated strong negative correlations between PAH concentrations in fish and increasing trophic levels, otherwise referred to as trophic- or bio-dilution^72,73^. Our analysis of 2,503 bile samples from 91 species of fish were all between trophic level three and five and demonstrated a very weak positive relationship with PAHs. This infers bile is a stronger indicator of recent exposure and biotransformation rates, whereas tissue concentrations of PAHs are a better indicator of assimilation rates and chronic accumulation.

## Implications

Biliary PAH metabolites in fish is a sensitive indicator of recent oil exposure (e.g., days to weeks) enabling the detection of long-term variation. In the north central GoM, biliary PAHs have demonstrated an increasing trend (108%) in oil exposure over time (2011–2017). This study determined an initial 24% decline in oil exposure the first three years post-DWH followed by a 173% increase by 2017 in the north central region of the GoM.

Species with the highest levels of PAH exposure were diverse, including pelagic, demersal and inshore species, indicating the PAH pollution is widespread throughout the Gulf ecosystem. Exposure to PAHs did not appear to be strongly associated with water temperature, depth, trophic level, habitat preference (pelagic vs benthic) or biometrics (i.e., length, weight). These data suggest habitat, lifestyle and biometrics may have relatively little consequence on PAH exposures due to the broadscale pollution in this region. The observed differences in biliary PAHs are likely functions of exposure frequencies, species-specific metabolism, diet, and feeding status. The highest concentrations of biliary TPAHeq in Yellowfin Tuna was a notable discovery. Identifying the potential sources in surface waters should be a focus of future research in order to protect pelagic species.

Historically, the northwest region of the Gulf has had the highest levels of biliary PAHs compared to other regions in the northern Gulf^74^. The highest levels of PAH exposure have now shifted to the north central region of the Gulf, adjacent to the Mississippi River Delta (Fig. 2). Fish with relatively high concentrations of biliary PAHs were collected in areas influenced by high volumes of riverine input, high densities of oil and gas infrastructure, previous spills and proximal to major harbors and navigation routes. The range of PAH exposures measured throughout the GoM is comparable to other polluted coastal waterways and areas immediately following oil spills. The region of the GoM with the lowest levels of PAH exposure was the Bay of Campeche, Mexico, a region with productive oil fields, active seeps and previous major spills. Based on the relatively low levels of biliary PAHs measured in fish from this region, perhaps this is a new baseline in which to measure recovery in fish collected in the northern GoM.

Our analyses provide the most comprehensive spatial and species baselines of PAH exposure ever conducted in the GoM and perhaps globally, although these baselines are not relevant in perpetuity. As noted, there have been significant temporal changes observed with both decreases and increases in contamination rates in the intervening 9+ years since DWH. The contamination rates have not changed monotonically since 2011 (e.g., post-DWH) indicating a complex interaction between continuing elevated exposures due to resuspended sedimented oil, continuing inputs from various other sources and the differential metabolic processes by the various fish species. We documented the progressive increases in PAH exposure in a number of GoM fishes, whereas PAH exposures in some species (e.g., sharks) are declining to an unknown and potentially shifting environmental baseline. The alarming increase in PAH exposure observed in multiple species should necessitate continued monitoring in the GoM. Furthermore, point sources need to be identified and reduced in order to reverse the directionality of this increasing trend in oil contamination to ensure the conservation of the GoM ecosystem.

**Figure 5:**
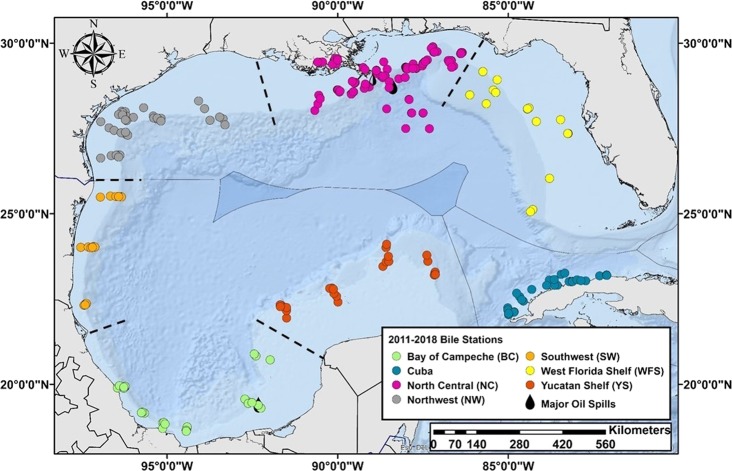
Locations of sampling stations for bile collections during the C-IMAGE comprehensive fish surveys in the GoM, 2011–2018. This figure was generated in ArcGIS Desktop Version 10.5.1.

Nevertheless, these Gulf-wide baselines will serve as a starting point for assessments of future large-scale contamination. Importantly, our sampling locations were not all immediately adjacent to the thousands of oil and gas wells located in the northern and southern GoM (Figs. 1 and 5). Thus, the information provided herein should be viewed as a general baseline for impact assessment and not for facility-specific assessment. Site-specific pre-impact assessments are necessary to accurately evaluate before-after, control, impact (BACI) effects. Given the enormous environmental impact of the *DWH* and *Ixtoc 1* spills it is perplexing that government oversight does not require such facility-specific baselines to be routinely collected as is required for other industries discharging into public waterways^75^.

## Methods

### Field collections

Between 2011 and 2018, we collected 15,026 specimens from 359 sampling stations using demersal and pelagic longline sampling gear along transects throughout the GoM (Fig. 5). Sampling design and protocols for the demersal longline efforts were previously described in detail^29^. Briefly, sampling efforts between 2011–2017 consisted of eight kilometers of demersal longline sets deployed along transects extending from shallow (40 m) to deep continental shelf areas (300 m). At each station, leader lines with 500–13/0 hooks were baited with cut fish (Atlantic Mackerel, *Scomber scombrus*) and squid (Humboldt squid wings, *Dosidicus gigas*) and clipped to the mainline. In 2018, 16 kilometers of pelagic longlines with an average of 159 baited 16/0 hooks were deployed at 16 stations in paired day-night sets in the top 200 m (mean hook depth of 68 m) in the north central region of the GoM. Temperature, time and depth recorders (Star-Oddi CDST Centi-TD, Gardabaer, Iceland) were attached to the beginning and end of all main line deployments. At the time of retrieval, all species and their biometrics were enumerated. A subsample of fishes was dissected and sampled for a variety of tissues, including blood, otoliths, eyes, muscle, liver, and bile. Bile was collected by first dissecting the liver and locating the gallbladder. If the specimen had a full gallbladder, it was carefully separated from the liver by cutting the bile duct and drained into a combusted amber vial. Samples were immediately frozen at −20 °C until analysis in the laboratory.

### Biliary PAH analysis

A total of 2,503 specimens collected between 2011 and 2018 were analyzed for biliary PAHs following guidelines and methodology recommended by the EPA, NOAA/NMFS and USGS^76–79^. Untreated bile samples (3 µL) from 91 species of fish and sharks were analyzed for naphthalene and benzo[*a*]pyrene metabolite equivalents using high performance liquid chromatography fluorescence detection (HPLC-F). Separations of PAH equivalents were achieved using a C18 reverse-phase column (Synergi 4 um Hydro-RP 80 Å, Phenomenex, Torrence, CA) set at 50 °C with a 1.0 mL min^−1^ gradient starting with 100% water with 0.5% Acetic Acid to 100% methanol (Optima LC/MS Grade). Fluorescence responses were measured using the excitation/emission wavelength pair for naphthalene (292/335 nm) and benzo[*a*]pyrene (380/430 nm) equivalents. All peaks within the PAH metabolite equivalent elution time window (5–19 minutes) were integrated and summed. Following previously validated methodology^26^, the fluorescence responses were converted to PAH metabolite equivalents, also known as fluorescent aromatic compounds (FACs), using naphthalene (2.5 µg mL^−1^) and benzo[*a*]pyrene (250 ng mL^−1^) external standards and the following formula:

$$Biliary\,PAH\,FACs=\frac{Standard\,concentration}{Mean\,Standard\,Area}\times \frac{integrated\,sample\,area}{bile\,density}\times \frac{volume\,of\,standard\,injected\,(10\,uL)}{volume\,of\,sample\,injected\,(3\,uL)}$$

where bile density is 1.03 g mL^−1^ ^76^.

On average, biliary PAH equivalents in all fish were comprised of 99.6 ± 2% naphthalene metabolites and less than 10% of benzo[*a*]pyrene (1.3 ± 8%) metabolite equivalents. The mean (± standard deviation) biliary PAH levels are reported as the sum of naphthalene and benzo[*a*]pyrene metabolite equivalents (TPAH_eq_ ng FAC g^−1^ bile) and rounded to two significant digits.

### Health proxies

Fulton’s condition factors (K) were calculated for each individual fish using the following formula:

$$K=\,100\,\times \frac{total\,weight\,(g)}{standard\,length\,{(cm)}^{3}}$$

where 100 is a scaling factor in order to bring K close to unity.

Hepato-somatic (HSI), gonadal-somatic (GoSI), gastrointestinal-somatic (GSI) indices were calculated for each individual fish following the formula:

$$HSI,\,GoSI\,or\,GSI=\frac{organ\,weight\,(g)}{total\,weight\,(g)}$$

where organ weight is the weight of the liver (HSI), gonads (GoSI) or gastrointestinal tract (GSI) for each individual fish.

### Quality assurance/quality control (QA/QC)

A quality assurance - quality control program following EPA^78^ and NOAA guidelines^76^ was implemented to monitor background contamination, precision and accuracy. Methanol blanks were analyzed prior to every standard and field sample to monitor carryover and background contamination. Any peaks found in the solvent blanks within the target elution window were subtracted from the field samples. All field samples were analyzed in triplicate. Any triplicates with a coefficient of variation (CV) greater than 20% were reanalyzed until QA/QC requirements were met (CV ≤ 20%). Continuing calibration standards for naphthalene and benzo[*a*]pyrene were analyzed every 12 field samples maintaining a CV ≤ 20%. Successful inter-laboratory calibration exercises were performed between Northwest Fisheries Science Center (NWFSC), Mote Marine Laboratory and the University of South Florida to confirm accurate concentrations for naphthalene and benzo[*a*]pyrene metabolite equivalents.

### Spatial modeling

Biliary PAHs in fish collected Gulf-wide from stations covering coastal, continental shelf and some deep-sea areas were included in the geostatistical analysis to generate a spatial PAH concentration map. Data were imported in ArcGIS Pro Version 2.3 (ESRI, Redlands, CA, USA) and the mean station values were interpolated using a “Kernel interpolation with barriers” method (Polynomial Order function). Spatial interpolation has been widely used for more than 50 years by environmental scientists, meteorologists, statisticians, geologists and GIS practitioners for mapping air pollution, earthquakes, habitat use and landscape patterns^80–82^. Kernel interpolation was used to allow for constraining the spatial analysis using a predefined “barrier”. Spatial interpolation predictions where then limited by the depth isobaths (10 to 1000 m) or by the outermost sampling stations. The interpolation was performed using a 2 km radius between any given sampling station. The interpolation maps were cross-validated for accuracy by calculating the mean prediction error (ME = [Σ_j=1,*n*_ *(x*^*^*^_*i*_ − *x*_*i*_)*/n*]) and the root-mean-square error (RMSE = sqrt[Σ_j=1,*n*_ *(x*^*^*^_*i*_ − *x*_*i*_*)*^2^ */n*]), where *x*^*^*^_*i*_ is the predicted value, *x*_*i*_ is the observed value, and *n* is the sample size. Ideal unbiased models should have an ME near 0 and an RSME close to 1^83^.

### Data analysis

The GoM was separated into seven regions *a priori* based on oceanographic features, benthic habitat types, and proximity to oil infrastructure (Fig. 5). The GoM regions identified were the Bay of Campeche (BC), Cuba (CUB), North Central (NC), Northwest (NW), Southwest (SW), West Florida Shelf (WFS), and the Yucatán Shelf (YS). Statistical analyses were performed using JMP Pro Version 14.3.0 (SAS Institute, Inc.) and MATLAB R2019b (Update 3, MathWorks) with the Fathom Toolbox^84^. Region, year, species and sex were determined to be significant variables effecting biliary TPAH concentrations in fish (all species combined) using permutation based model effects and Akaike’s Information Criteria (AIC) (Table S1). Interaction effects were not further evaluated due to the unbalanced sampling design in all variables. The differences in mean biliary PAHs between regions and years were assessed using a modified permutational multivariate analysis of variance (PERMANOVA) and pair-wise modified PERMANOVA with 1,000 permutations and an α = 0.05. These are semiparametric methods that use the appropriate distribution-free permutation techniques accounting for unbalanced/asymmetrical designs and heterogenous dispersions among groups^85^. Species differences were evaluated using the Games-Howell Honestly Significant Difference (HSD) approach designed for unequal variances and sample sizes. Biometrics (i.e., total weight, standard length, somatic indices) were standardized by species using z-scores prior to statistical analysis^86^. Pearson’s correlations and regressions were used to explore relationships between PAHs and variables (e.g., standard length z-scores, temperature etc.).

## Supplementary information

Supplementary Information.

## Data Availability

Data are publicly available through the GoM Research Initiative Information and Data Cooperative (GRIIDC) at https://data.gulfresearchinitiative.org (doi: 10.7266/N7X34W1J).
