# Supplementary material for: A First Comprehensive Baseline of Hydrocarbon Pollution in Gulf of Mexico Fishes
Source: Sci Rep. 2020 Apr 15;10:6437. doi: 10.1038/s41598-020-62944-6 (PMC7160155; doi:10.1038/s41598-020-62944-6)
Supplement: Supplementary file 1 — Supplementary Information. [file 41598_2020_62944_MOESM1_ESM.pdf]

A First Comprehensive Baseline of Hydrocarbon Pollution in Gulf of Mexico Fishes

Erin L. Pulster[a*], Adolfo Gracia[b], Maickel Armenteros[b,c], Gerardo Toro-Farmer[d], Susan M. Snyder[a], Brigid E. Carr[a], Madison R. Schwaab[a], Tiffany J. Nicholson[a], Justin Mrowicki[a], Steven A. Murawski[a]

[a]University of South Florida, 140 7th Avenue South, St. Petersburg, FL 33701 USA
[b]Universidad Nacional Autónoma de México, Instituto de Ciencias del Mar y Limnología, Ciudad de México, CDMX, México
[c]Universidad de La Habana, Centro de Investigaciones Marinas, 16 # 114, Playa, Habana, 11300, Cuba
[d]New College of Florida, 5800 Bay Shore Road, Sarasota, FL, 34243 USA

Supplemental Information

Table S1. Main parameter effects on biliary TPAHeq concentrations in Gulf of Mexico fishes collected between 2011 and 2018 using permutation based model effects and Akaike's Information Criteria (AIC).

Table S2. Statistically significant (Games-Howell HSD post-hoc comparisons) species comparisons of log transformed biliary PAH (µg PAH FAC g-1 bile) collected in the Gulf of Mexico (2011-2018).

Table S1. Main parameter effects on biliary TPAHeq concentrations in Gulf of Mexico fishes collected between 2011 and 2018 using permutation based model effects and Akaike's Information Criteria (AIC).

**Variable**	**RSS**	**AIC**	**Ratio**	**Permutation-*p***
Region	7.6151e+13	6.0423e+04	1	0.002
Species	7.6314e+13	6.0428e+04	14.5438	0.002
Year	7.8738e+13	6.0506e+04	1.4455e+18	0.002
Sex	7.9008e+13	6.0515e+04	1.0483e+20	0.002

Table S2. Statistically significant (Games-Howell HSD post-hoc comparisons) species comparisons of log transformed biliary PAH (µg PAH FAC g-1 bile) collected in the Gulf of Mexico (2011-2018).

Level	-Level	DF	LCL	UCL	*p*-Value
Alligator Gar	Great Barracuda	7.287066396	0.805730293	2.747500344	*0.0005*
Alligator Gar	Speckled Hind	13.13075382	0.565332673	2.9165869	*0.0006*
Alligator Gar	Blackline Tilefish	12.57955803	0.458304269	2.32269513	*0.0006*
Alligator Gar	Coney	5.590945277	1.108814583	3.245790305	*0.0007*
Alligator Gar	Greater Amberjack	7.947585805	0.618102902	2.522584343	*0.0009*
Alligator Gar	Red Hind	6.844049541	0.662050763	2.585894472	*0.0011*
Alligator Gar	Red Porgy	5.803767096	0.702698789	2.710519121	*0.0016*
Alligator Gar	Spineycheek Scorpionfish	16.96374099	0.397179296	3.76628362	*0.0028*
Alligator Gar	Yellowedge Grouper	3.321989195	1.222825781	3.786259667	*0.0029*
Alligator Gar	Silky Shark	5.999998185	0.534151498	3.613881601	*0.0053*
Alligator Gar	Gulf Hake	3.152409077	0.869319844	3.526679591	*0.0054*
Alligator Gar	Wenchman	3.95967901	0.458844666	2.774974586	*0.0072*
Alligator Gar	Yellowtail Snapper	14.79223758	0.125459366	3.078635082	*0.012*
Alligator Gar	Vermilion Snapper	13.18185355	0.096683159	2.456474482	*0.0129*
Alligator Gar	Smooth Dogfish	6.732121438	0.277156914	4.676989019	*0.013*
Alligator Gar	Crevalle Jack	2.941840061	0.343188954	3.721286655	*0.0148*
Alligator Gar	King Snake Eel	2.205829435	0.360360054	4.051159935	*0.017*
Alligator Gar	Palespotted Eel	10.75961593	0.006447468	4.557492176	*0.0245*
Alligator Gar	Blueline Tilefish	3.997512936	-0.049021108	3.131995865	*0.028*
Alligator Gar	Barrelfish	3.861707899	-0.160730445	2.735410236	*0.0378*
Alligator Gar	Bearded Brotula	5.247385016	-0.397993603	8.077728848	*0.0388*
Alligator Gar	Black Grouper	2.960004379	-0.380019048	3.640530717	*0.0449*
Alligator Gar	Longnose Lancetfish	6.513471235	-0.128565476	1.975586117	*0.0488*
Alligator Gar	Gafftopsail Catfish	21.13074598	1.965803866	4.613064058	*<.0001*
Alligator Gar	Scamp	20.96925766	1.540309789	3.950816664	*<.0001*
Alligator Gar	Red Grouper	12.48248937	1.387725363	3.22953966	*<.0001*
Alligator Gar	Southern Hake	11.62510814	1.319225435	3.185272169	*<.0001*
Alligator Gar	Little Gulper Shark	19.16843216	0.961130986	3.413636531	*<.0001*
Alligator Gar	Snowy Grouper	13.12346901	1.00887479	3.053888342	*<.0001*
Alligator Gar	Almaco Jack	16.95261423	0.912624528	2.962687568	*<.0001*
Alligator Gar	Silk Snapper	12.54078298	0.737064896	2.610486529	*<.0001*
Almaco Jack	Gafftopsail Catfish	41.40256192	-0.059552191	2.763108019	*0.0423*
Barrelfish	Gafftopsail Catfish	23.90397385	0.637227619	3.366960514	*0.0001*
Barrelfish	Scamp	23.87762134	0.204486204	2.711960458	*0.0042*
Barrelfish	Yellowedge Grouper	5.951020005	0.152741743	2.281663913	*0.0127*
Barrelfish	Red Grouper	15.35290527	0.033414913	2.009170319	*0.0186*
Barrelfish	Southern Hake	14.53282336	-0.030286141	1.960103954	*0.0324*
Black Drum	Gafftopsail Catfish	10.07700926	0.233706665	4.994637604	*0.0127*
Black Grouper	Gafftopsail Catfish	18.64774748	0.198334055	3.1200222	*0.0067*
Blackfin Snapper	Gafftopsail Catfish	24.48624905	-0.121175416	4.022703399	*0.0465*
Blackfin Tuna	Scamp	28.7487955	0.517659161	3.285943569	*0.0003*
Blackfin Tuna	Yellowedge Grouper	11.15629131	0.518539955	2.803021769	*0.0011*
Blackfin Tuna	Red Grouper	19.61534246	0.299048202	2.630693098	*0.0019*
Blackfin Tuna	Southern Hake	18.99575443	0.239743151	2.57723073	*0.0033*
Blackfin Tuna	Gulf Hake	10.96152983	0.211188407	2.497287305	*0.0066*

Level	-Level	DF	LCL	UCL	*p*-Value
Blackfin Tuna	King Snake Eel	9.798859615	0.211058707	2.512937559	*0.0073*
Blackfin Tuna	Coney	13.5188086	0.166417169	2.500663995	*0.0078*
Blackfin Tuna	Snowy Grouper	20.82233389	-0.042069243	2.417308652	*0.0352*
Blackfin Tuna	Crevalle Jack	9.855244582	-0.07103985	2.447991735	*0.037*
Blackfin Tuna	Little Gulper Shark	27.08384975	-0.052828531	2.740072323	*0.038*
Blackfin Tuna	Gafftopsail Catfish	29.073076	0.966899694	3.924444507	<*.0001*
Blackline Tilefish	King Snake Eel	66.94048805	0.1252348	1.505285789	*0.0015*
Blackline Tilefish	Gulf Hake	78.03008521	0.093486869	1.521513167	*0.0033*
Blackline Tilefish	Scamp	43.94130298	0.134106356	2.576020698	*0.0057*
Blackline Tilefish	Red Grouper	113.8200823	0.046456325	1.789809299	*0.0108*
Blackline Tilefish	Southern Hake	93.98063309	-0.012500689	1.735998894	*0.0308*
Blackline Tilefish	Coney	45.64740179	-0.039395492	1.613000981	*0.0458*
Blackline Tilefish	Gafftopsail Catfish	37.02586863	0.557094235	3.240774291	<*.0001*
Blackline Tilefish	Yellowedge Grouper	79.62657331	0.395989005	1.832097044	<*.0001*
Blueline Tilefish	Gafftopsail Catfish	21.65073917	0.309194828	3.18669834	*0.0026*
Crevalle Jack	Gafftopsail Catfish	22.72973902	-0.084741611	2.599133927	*0.0479*
Dolphinfish	Scamp	38.711257	0.572053384	3.486425734	*0.0001*
Dolphinfish	Yellowedge Grouper	20.19018946	0.610950915	2.965487199	*0.0001*
Dolphinfish	Red Grouper	30.55613492	0.351405716	2.833211973	*0.0008*
Dolphinfish	Southern Hake	29.71501048	0.294028403	2.777821867	*0.0014*
Dolphinfish	King Snake Eel	18.41623208	0.320036264	2.65883639	*0.0018*
Dolphinfish	Gulf Hake	19.93855503	0.305635029	2.657717072	*0.0018*
Dolphinfish	Coney	22.76532484	0.242960653	2.6789969	*0.0029*
Dolphinfish	Crevalle Jack	17.07601241	0.047506726	2.584321549	*0.0177*
Dolphinfish	Snowy Grouper	31.06912564	0.013052003	2.617063795	*0.0222*
Dolphinfish	Little Gulper Shark	36.53253096	0.00414603	2.937974152	*0.0241*
Dolphinfish	Gafftopsail Catfish	37.96474958	1.026540477	4.119680114	<*.0001*
Golden Tilefish	Speckled Hind	15.35808208	0.618563541	2.74256646	*0.0002*
Golden Tilefish	Spineycheek Scorpionfish	16.59869738	0.374248579	3.668424765	*0.0031*
Golden Tilefish	Longnose Lancetfish	12.57631183	0.142943471	1.5832876	*0.0053*
Golden Tilefish	Silky Shark	5.507867814	0.505997117	3.521246411	*0.0061*
Golden Tilefish	Vermilion Snapper	15.34293104	0.148369342	2.283998727	*0.0074*
Golden Tilefish	Yellowtail Snapper	14.74727417	0.118539249	2.964765628	*0.0122*
Golden Tilefish	Crevalle Jack	2.712889946	0.429886673	3.513799364	*0.0131*
Golden Tilefish	Smooth Dogfish	6.236324438	0.198088609	4.635267754	*0.016*
Golden Tilefish	Barrelfish	3.782849276	-0.048077272	2.501967492	*0.0286*
Golden Tilefish	Palespotted Eel	10.25076746	-0.046319717	4.48946979	*0.0289*
Golden Tilefish	Blueline Tilefish	3.54662499	-0.089761158	3.051946344	*0.0303*
Golden Tilefish	Bearded Brotula	5.065042736	-0.506688394	8.065634067	*0.043*
Golden Tilefish	Gafftopsail Catfish	23.28307047	1.977836369	4.480241985	<*.0001*
Golden Tilefish	Scamp	25.74009104	1.57388617	3.796450712	<*.0001*
Golden Tilefish	Yellowedge Grouper	242.6019015	2.067378818	2.820917058	<*.0001*
Golden Tilefish	Red Grouper	82.03600817	1.602140568	2.894334883	<*.0001*
Golden Tilefish	Southern Hake	53.68376476	1.534909635	2.848798398	<*.0001*
Golden Tilefish	King Snake Eel	522.6239642	1.836816644	2.453913774	<*.0001*
Golden Tilefish	Gulf Hake	284.2256473	1.770266316	2.504943548	<*.0001*
Golden Tilefish	Little Gulper Shark	22.51712878	0.991445576	3.262532369	<*.0001*
Golden Tilefish	Coney	13.16480662	1.463530447	2.77028487	<*.0001*
Golden Tilefish	Snowy Grouper	22.75779584	1.132050445	2.809923116	<*.0001*

Level	-Level	DF	LCL	UCL	*p*-Value
Golden Tilefish	Almaco Jack	31.30110302	1.014258075	2.74026445	*<.0001*
Golden Tilefish	Great Barracuda	35.89984211	1.137943705	2.29449736	*<.0001*
Golden Tilefish	Red Porgy	68.16907765	1.151178057	2.141250282	*<.0001*
Golden Tilefish	Silk Snapper	51.91927947	0.934201201	2.292560653	*<.0001*
Golden Tilefish	Red Hind	114.4281477	1.05266747	2.074488194	*<.0001*
Golden Tilefish	Wenchman	113.8519224	1.142391633	1.970638049	*<.0001*
Golden Tilefish	Greater Amberjack	48.14835005	0.934080397	2.085817277	*<.0001*
Golden Tilefish	Blackline Tilefish	57.91399905	0.659422041	2.000787787	*<.0001*
Golden Tilefish	Red Snapper	934.3212763	0.980668994	1.518703564	*<.0001*
Great Barracuda	Yellowedge Grouper	56.29442986	0.099327488	1.356527323	*0.0025*
Great Barracuda	Gafftopsail Catfish	31.39626065	0.205652799	2.819984489	*0.0036*
Greater Amberjack	Gafftopsail Catfish	32.18791453	0.409686301	3.028494379	*0.0005*
Greater Amberjack	King Snake Eel	58.68142933	0.037756445	1.233076298	*0.0102*
Greater Amberjack	Gulf Hake	71.7004106	0.002681423	1.252630768	*0.0235*
Greater Amberjack	Scamp	37.56027265	-0.007252502	2.357691709	*0.027*
Greater Amberjack	Yellowedge Grouper	73.50599637	0.304582015	1.563816187	*<.0001*
Little Tunny	Gafftopsail Catfish	24.07029522	0.532299933	3.798187307	*0.0007*
Little Tunny	Scamp	22.23556475	0.061135802	3.181609966	*0.0166*
Little Tunny	Yellowedge Grouper	8.849900537	-0.070598003	2.831302766	*0.0344*
Longnose Lancetfish	Coney	19.81481342	0.4218507	2.085733546	*0.0002*
Longnose Lancetfish	Snowy Grouper	29.93905584	0.134430131	2.08131236	*0.0048*
Longnose Lancetfish	Great Barracuda	28.22887924	0.045037655	1.66117234	*0.0132*
Longnose Lancetfish	Crevalle Jack	8.110808679	0.075712852	2.141742115	*0.0161*
Longnose Lancetfish	Little Gulper Shark	29.73201224	0.04361261	2.484134265	*0.0165*
Longnose Lancetfish	Almaco Jack	37.36242662	0.015155705	2.01313575	*0.0207*
Longnose Lancetfish	Red Porgy	26.74297576	0.012351942	1.553845327	*0.0209*
Longnose Lancetfish	Wenchman	21.58402133	-0.048512644	1.435311254	*0.0484*
Longnose Lancetfish	Gafftopsail Catfish	29.95159021	1.040609682	3.6912376	*<.0001*
Longnose Lancetfish	Scamp	33.27125574	0.619326147	3.024779663	*<.0001*
Longnose Lancetfish	Yellowedge Grouper	19.55945194	0.848782177	2.313282629	*<.0001*
Longnose Lancetfish	Red Grouper	43.33863014	0.532429566	2.237814815	*<.0001*
Longnose Lancetfish	Southern Hake	38.66261931	0.471438246	2.186038716	*<.0001*
Longnose Lancetfish	King Snake Eel	15.31250062	0.560647533	2.003851814	*<.0001*
Longnose Lancetfish	Gulf Hake	18.95416287	0.544286868	2.004691925	*<.0001*
Queen Snapper	Scamp	31.48315384	0.346509397	3.14862892	*0.0012*
Queen Snapper	Yellowedge Grouper	13.49014624	0.367829651	2.645267661	*0.0019*
Queen Snapper	Red Grouper	22.59913159	0.130213023	2.491063864	*0.0077*
Queen Snapper	Southern Hake	21.90663308	0.071598958	2.436910511	*0.0133*
Queen Snapper	King Snake Eel	11.99689723	0.067928422	2.347603431	*0.0158*
Queen Snapper	Gulf Hake	13.27669926	0.061405402	2.338605898	*0.0161*
Queen Snapper	Coney	15.94353083	0.007599778	2.351016975	*0.0236*
Queen Snapper	Gafftopsail Catfish	31.4876141	0.796792714	3.786087075	*<.0001*
Red Drum	Speckled Hind	30.07642477	0.468221279	2.801149003	*0.0002*
Red Drum	Silky Shark	12.13838139	0.604683545	3.330800263	*0.001*
Red Drum	Spineycheek Scorpionfish	23.42201311	0.27504209	3.675871535	*0.0043*
Red Drum	Blueline Tilefish	11.48333143	0.251655736	2.618769729	*0.0052*
Red Drum	Barrelfish	15.25389841	0.15963891	2.202491591	*0.0064*
Red Drum	Black Grouper	8.602774047	0.250826383	2.797135995	*0.0075*
Red Drum	Smooth Dogfish	9.099863485	0.284300136	4.457296506	*0.0104*

Level	-Level	DF	LCL	UCL	*p*-Value
Red Drum	Yellowtail Snapper	23.31731359	0.013843776	2.977701381	*0.0226*
Red Drum	Vermilion Snapper	29.92666112	-0.000936632	2.341544981	*0.0252*
Red Drum	Misty Grouper	4.959883554	-0.06807642	3.926356551	*0.029*
Red Drum	Palespotted Eel	13.17787587	-0.07755163	4.428941983	*0.0331*
Red Drum	Bearded Brotula	5.818775925	-0.369454985	7.836640938	*0.0393*
Red Drum	Cuban Dogfish	5.492760499	-0.169006219	3.434294045	*0.0393*
Red Drum	Gafftopsail Catfish	36.76376242	1.82837831	4.537940323	*<.0001*
Red Drum	Scamp	42.77627057	1.402719525	3.875857637	*<.0001*
Red Drum	Yellowedge Grouper	46.31403551	1.634409871	3.162126286	*<.0001*
Red Drum	Red Grouper	77.98465655	1.300618393	3.104097339	*<.0001*
Red Drum	Southern Hake	68.40012862	1.241721816	3.050226497	*<.0001*
Red Drum	King Snake Eel	38.93820784	1.358918128	2.840052569	*<.0001*
Red Drum	Gulf Hake	45.31276074	1.331287306	2.852162838	*<.0001*
Red Drum	Little Gulper Shark	38.02651737	0.829397568	3.332820656	*<.0001*
Red Drum	Coney	37.06434849	1.21006694	2.931988656	*<.0001*
Red Drum	Crevalle Jack	14.21240591	0.941738472	2.910187845	*<.0001*
Red Drum	Snowy Grouper	45.2300489	0.913807053	2.936406788	*<.0001*
Red Drum	Almaco Jack	54.97120391	0.791028643	2.871734162	*<.0001*
Red Drum	Great Barracuda	54.33975674	0.820409397	2.520271948	*<.0001*
Red Drum	Red Porgy	56.1250653	0.787870115	2.412798504	*<.0001*
Red Drum	Silk Snapper	69.01872632	0.649087651	2.485914483	*<.0001*
Red Drum	Red Hind	63.10758	0.693823296	2.341572647	*<.0001*
Red Drum	Wenchman	49.27283796	0.733303503	2.287966458	*<.0001*
Red Drum	Greater Amberjack	59.59954659	0.611117615	2.317020339	*<.0001*
Red Drum	Blackline Tilefish	71.50105774	0.369997996	2.198452112	*<.0001*
Red Drum	Red Snapper	35.29629919	0.474460253	1.933152586	*<.0001*
Red Hind	Gafftopsail Catfish	31.00031889	0.369208819	2.961713871	*0.0007*
Red Hind	King Snake Eel	141.5033828	0.043407418	1.120167335	*0.0067*
Red Hind	Gulf Hake	168.8887091	0.002641746	1.145412455	*0.0232*
Red Hind	Scamp	36.25557323	-0.044859363	2.28804058	*0.0397*
Red Hind	Yellowedge Grouper	170.5246916	0.303692321	1.457447892	*<.0001*
Red Porgy	Gafftopsail Catfish	29.72347945	0.292618767	2.873031247	*0.0016*
Red Porgy	King Snake Eel	87.00025677	-0.022851229	1.021153307	*0.0466*
Red Porgy	Yellowedge Grouper	110.6803413	0.237454979	1.358412558	*<.0001*
Red Snapper	Scamp	26.21609871	0.321917285	2.549047038	*0.0009*
Red Snapper	Coney	14.0846195	0.213634559	1.520808199	*0.0017*
Red Snapper	Gafftopsail Catfish	23.63267692	0.72630929	3.232396505	*<.0001*
Red Snapper	Yellowedge Grouper	270.0188169	0.806892389	1.58203093	*<.0001*
Red Snapper	Red Grouper	85.69653291	0.346593405	1.650509488	*<.0001*
Red Snapper	Southern Hake	56.12953946	0.279853157	1.604482318	*<.0001*
Red Snapper	King Snake Eel	586.7901129	0.573759683	1.217598176	*<.0001*
Red Snapper	Gulf Hake	316.9415341	0.509452577	1.266384729	*<.0001*
Silk Snapper	Gafftopsail Catfish	36.9902021	0.271878796	2.959437704	*0.0018*
Silk Snapper	Yellowedge Grouper	71.39102086	0.105631233	1.55590279	*0.0027*
Skipjack Tuna	Gafftopsail Catfish	5.878304426	1.216598596	5.997846658	*0.0029*
Skipjack Tuna	Scamp	4.768271231	0.527255937	5.599447844	*0.0111*
Skipjack Tuna	Spineycheek Scorpionfish	8.534614095	0.058870943	4.740169302	*0.0213*
Skipjack Tuna	Smooth Dogfish	5.778358105	0.055987329	5.533735933	*0.0227*
Skipjack Tuna	Bearded Brotula	5.931593869	-0.025877697	8.34119027	*0.0258*

Level	-Level	DF	LCL	UCL	*p*-Value
Skipjack Tuna	Little Gulper Shark	4.743591453	-0.041977242	5.052322087	*0.0268*
Skipjack Tuna	Palespotted Eel	9.202152766	-0.042025017	5.24154199	*0.0278*
Speckled Hind	Gafftopsail Catfish	35.980736	0.068415957	3.028532394	*0.0141*
Swordfish	Scamp	20.02975166	0.435332829	3.591911848	*0.0015*
Swordfish	Yellowedge Grouper	7.627835322	0.252398174	3.292805498	*0.0098*
Swordfish	Red Grouper	11.92059682	0.111134743	3.042248504	*0.014*
Swordfish	Southern Hake	11.71214098	0.05134508	2.989270748	*0.0192*
Swordfish	Gulf Hake	7.528522126	-0.057391126	2.989508786	*0.0312*
Swordfish	Coney	9.145637618	-0.053619994	2.944343106	*0.0317*
Swordfish	King Snake Eel	6.934849046	-0.072712779	3.020350992	*0.0325*
Swordfish	Gafftopsail Catfish	21.97904192	0.910122516	4.204863633	<*.0001*
Vermilion Snapper	Gafftopsail Catfish	35.98854557	0.529749344	3.495960941	*0.0002*
Vermilion Snapper	Yellowedge Grouper	18.68736143	0.149611071	2.306316736	*0.0065*
Vermilion Snapper	Scamp	37.35350853	0.082684397	2.855284416	*0.0118*
Wenchman	Gafftopsail Catfish	27.32048208	0.3983043	2.946744372	*0.0006*
Wenchman	Gulf Hake	184.9378328	0.093351178	1.068829005	*0.0008*
Wenchman	Scamp	31.22775786	-0.011051715	2.268358915	*0.028*
Wenchman	Red Grouper	114.7738517	-0.022298274	1.405744045	*0.0397*
Wenchman	Yellowedge Grouper	183.8029982	0.393280429	1.381985766	<*.0001*
Wenchman	King Snake Eel	152.9988039	0.140976881	1.036723855	<*.0001*
Yellowfin Tuna	Gafftopsail Catfish	13.15617036	1.36906338	6.510323707	*0.0004*
Yellowfin Tuna	Scamp	11.96972785	0.834706853	5.956938763	*0.0022*
Yellowfin Tuna	Yellowedge Grouper	7.557108457	0.504600655	5.805003955	*0.0087*
Yellowfin Tuna	Smooth Dogfish	12.61819511	0.356238165	5.898426931	*0.0091*
Yellowfin Tuna	Bearded Brotula	9.905960239	0.551100564	8.429153843	*0.0097*
Yellowfin Tuna	Red Grouper	8.89805687	0.36646491	5.551319276	*0.0102*
Yellowfin Tuna	Little Gulper Shark	11.93645154	0.273399197	5.401887482	*0.0111*
Yellowfin Tuna	Southern Hake	8.85548701	0.308196407	5.49682036	*0.0118*
Yellowfin Tuna	Coney	8.117060747	0.203442665	5.451681386	*0.0157*
Yellowfin Tuna	Gulf Hake	7.524159578	0.196127859	5.500390739	*0.0164*
Yellowfin Tuna	King Snake Eel	7.324996651	0.191541453	5.520497698	*0.0167*
Yellowfin Tuna	Silky Shark	9.919429823	0.110201732	5.33835053	*0.0187*
Yellowfin Tuna	Spineycheek Scorpionfish	15.85451395	0.082220129	5.381761949	*0.0189*
Yellowfin Tuna	Snowy Grouper	9.561907258	0.101599744	5.261682551	*0.0192*
Yellowfin Tuna	Palespotted Eel	16.27163935	0.059124876	5.80533393	*0.0207*
Yellowfin Tuna	Crevalle Jack	7.889978294	0.038871402	5.32612337	*0.0229*
Yellowfin Tuna	Almaco Jack	10.03786807	0.018056476	5.157774783	*0.0238*
Yellowfin Tuna	Misty Grouper	8.959413306	-0.026421045	5.397769629	*0.0266*
Yellowfin Tuna	Shortfin Mako	10.50846894	-0.165221624	6.163339903	*0.0364*
Yellowfin Tuna	Great Barracuda	8.269685221	-0.188783759	5.042533558	*0.0394*
Yellowfin Tuna	Speckled Hind	10.55756338	-0.182774139	4.965212875	*0.0418*
Yellowfin Tuna	Yellowmouth Grouper	10.21816079	-0.394328045	8.434721204	*0.0473*
Yellowfin Tuna	Red Porgy	7.993745052	-0.270568695	4.984305768	*0.0473*
Yellowfin Tuna	Silk Snapper	8.990355485	-0.266311351	4.914381939	*0.0495*
Yellowtail Snapper	Gafftopsail Catfish	33.50518599	-0.014175318	3.388948794	*0.0276*
